# Commemoration of the Dead in the Time of COVID-19

**DOI:** 10.1017/dmp.2021.52

**Published:** 2021-02-16

**Authors:** Jeff Clyde G. Corpuz

**Affiliations:** Department of Theology and Religious Education, De La Salle University, Manila, Philippines

**Keywords:** COVID-19, commemoration, dead, crisis, public health

*The life of the dead is placed in the memory of the living.*

Marcus Tullius Cicero

The emergence of the coronavirus disease 2019 (COVID-19) pandemic has had a profound impact on many aspects of dying, death, burials, cremation, and funerals. Indeed, disasters and pandemics have long-lasting effects due to the high mortality rate, highlighting the importance of crisis management strategies and coping mechanisms.

Although little attention has been paid to the dead in times of crisis, the concept of commemoration of the dead has been largely overlooked and remains unexplored. Studies acknowledge that the commemoration of the dead is greatly inspired by religious,^1^ cultural, and sociological factors.^2^ Overall, by linking the commemoration of the dead in the context of pandemic and disaster medicine and public health preparedness, this research contributes to knowledge advancement on respecting the dead in the context of a pandemic.

## Rituals for the Dead

The COVID-19 pandemic presents challenges in funeral practices. However, evidence from previous pandemics suggests that people are willing to adapt practices, “provided the new practices meet the symbolic, social and emotional needs of the original ceremonies and practices, and affected communities themselves are involved in the formulation of any proposed changes.”^2^ The World Health Organization^3^ suggested key considerations for events related to death, burial, funeral (rites, ceremonies, and practices), and mourning in the context of the COVID-19 pandemic.

## Online Memorials

In societies where mass gatherings are not allowed, bereaved families are encouraged to explore alternatives. Social media platforms such as Facebook, Instagram, Twitter, and Zoom have become “online obituaries.” New settings, both temporal and spiritual, are accommodated to mitigating the grief, pain, and loss of loved ones due to COVID-19. In many settings, the bereaved may explore creative ways, such as “virtual memorial services, live streaming, and online eulogies” or “cybermemorials (also called Web memorials) and virtual cemeteries.”^4^ Memorializing those who have died in this time of crisis can be a very difficult and complex process. The *New York Times* on May 24, 2020, gave their full front page in remembrance of the near 100,000 deaths in the United States.^5^

## Intercultural and Interreligious Considerations

Considering intercultural and interreligious dimensions of the pandemic, sensitivities should be properly addressed. In many countries, cremation has been enforced. For instance, China and South Korea imposed mandatory cremation as a response to mitigate the epidemics. This precedent may present challenges to other religions that do not practice cremation, such as Islam and Judaism. Although, cremation can be positively received by cultures and religions that generally accept cremation, such as Buddhism, Hinduism, and Christianity. The Centers for Disease Control and Prevention (CDC) has not made cremations mandatory during COVID-19 and families have the freedom to choose between burial or cremation.

The COVID-19 pandemic has affected all people. Perhaps, one great realization that people should learn from the COVID-19 pandemic is the reality of death. Post COVID-19 research is needed to study the coping mechanisms of bereaved family members as a result of the death of family members. Due to cultural and religious differences that are traditionally connected to death, dying, and funeral practices, banning funeral ceremonies may have severe psychological consequences for the family members who are unable to perform burial rituals. However, “virtual memorials” may lead to a delayed grief process that could lead to long-term physiological and emotional difficulties. Nevertheless, it may be helpful to encourage family members to rethink the old ways of providing rituals for the dead. Commemoration of the death in times of COVID-19 has become a difficult and complex event.
